# Relationship between perceived school climate and adolescent aggressive behavior: a moderated mediation model

**DOI:** 10.3389/fpsyg.2026.1791314

**Published:** 2026-06-08

**Authors:** Liying Wen, Fei He, Yongzhi Jiang, Lifang Tong

**Affiliations:** 1 Inner Mongolia Autonomous Region, Inner Mongolia University for Nationalities, School of Educational Science, Tongliao, China; 2 Research Center for Student Bullying Prevention and Control in Inner Mongolia Autonomous Region, Tongliao, China

**Keywords:** adolescents, aggressive behavior, friendship quality, psychological resilience, school climate

## Abstract

**Objective:**

Examine how perceived school climate influences adolescent aggressive behavior, focusing on the mediating role of friendship quality and the moderating role of psychological resilience using a moderated mediation model grounded in ecological systems theory, interpersonal perspectives, and resilience frameworks.

**Methods:**

A cross-sectional survey was conducted among 4991 students (aged 8–20 years) from 36 primary (Grades 4 and 5), middle school (Grades 1 and 2), and high schools (Grades 10 and 11) in the Inner Mongolia Autonomous Region, China. Schools were selected using purposive sampling, and classes within schools were randomly sampled. Data were collected via self-report questionnaires, including the Perceived School Climate Scale, Aggressive Behavior Scale, Friendship Quality Scale, and Psychological Resilience Scale.

**Results:**

Perceived school climate, friendship quality, and psychological resilience were all significantly and positively correlated. Aggressive behavior was significantly negatively correlated with perceived school climate, friendship quality, and psychological resilience. Friendship quality significantly mediated the relationship between perceived school climate and aggressive behavior. Additionally, psychological resilience significantly moderated the effects of both perceived school climate and friendship quality on aggressive behavior.

**Conclusion:**

Perceived school climate is associated with adolescent aggressive behavior both directly and indirectly through friendship quality, although the mediating effect is modest. This study contributes to the literature by integrating environmental, interpersonal, and individual factors within a single framework and by providing empirical evidence from an understudied regional context in China. Additionally, psychological resilience emerges as a protective factor that mitigates the negative associations between school climate, friendship quality, and aggressive behavior.

## 1 Introduction

Aggressive behavior has become a major concern for schools worldwide (Hymel and Swearer, 2015). A growing body of research indicates that youth aggression, including bullying, verbal hostility, and physical violence, remains prevalent across diverse cultural contexts and poses significant challenges for educational systems and public health

(Zhang et al., 2022; Malti et al., 2015). According to UNESCO (2019), approximately one in three children globally has experienced bullying, highlighting the widespread nature of aggressive behavior in school settings. In addition to global evidence, aggressive behavior among adolescents has also been identified as a serious issue in China (Song et al., 2025), where both individual and contextual factors contribute to its development (Zhang et al., 2022). Within China, these processes may vary across regions. The Inner Mongolia Autonomous Region, characterized by a multi-ethnic composition and shaped by collectivist cultural values, may involve diverse norms of peer interaction and conflict resolution (Zhang, 2026).

Aggressive behavior refers to physical or psychological actions intended to harm others, encompassing hostile, harmful, or destructive acts expressed through physical, verbal, or relational means (Li and Si, 2024). Among adolescents, such behaviors often manifest as physical aggression, verbal abuse, psychological intimidation, or social exclusion (Estévez et al., 2013). These behaviors not only undermine peer relationships but also disrupt the overall school environment and hinder adolescents' social and emotional development (Malti et al., 2015; Kuzhiyengal Mambra and Kotian, 2024). Consequently, reducing adolescent aggression is essential for fostering healthy development and maintaining a positive educational environment.

Previous research suggests that aggressive behavior is influenced by multiple factors, including school (Bui and Nguyen, 2024) individual characteristics (e.g., emotional regulation), family dynamics, and broader social contexts (Zhang et al., 2022). Among these, the school environment plays a particularly critical role during adolescence, as schools become the primary setting for social interaction and development (Gettinger, 2003; Thapa et al., 2013). A growing body of literature highlights that a positive school climate can promote a sense of belonging, safety, and emotional support, thereby reducing aggressive tendencies (Da'as, 2025; Wang et al., 2017; Johnson, 2009). Conversely, negative school environments characterized by conflict, lack of support, or perceived injustice may contribute to psychological distress and increase aggressive behavior (Zhao et al., 2021; Konishi et al., 2017). In regions such as Inner Mongolia, where boarding schools are common due to geographic dispersion, adolescents may spend extended periods in school settings, potentially amplifying the influence of school climate on behavior (Shen and Wang, 2025; Yu, 2025).

At the same time, contemporary research suggests that adolescent peer relationships are evolving in the digital age, where online communication and media exposure increasingly shape friendship patterns and social interactions (Nesi et al., 2018). These changes may influence how peer relationships function as protective or risk factors for aggressive behavior (Prinstein and Giletta, 2020). Additionally, in multi-ethnic settings such as Inner Mongolia (Zhang, 2026), processes of inter-ethnic peer acceptance and communication between Mongolian and Han students may shape how friendships develop and function, potentially influencing this mediating pathway. However, empirical studies integrating school climate, evolving peer dynamics, and individual psychological characteristics remain limited. Therefore, this study seeks to examine the impact of perceived school climate on adolescent aggressive behavior, with particular attention to the underlying interpersonal and individual mechanisms.

### 1.1 Adolescents' perceived school climate and aggressive behavior

According to ecological systems theory, adolescents' development is shaped by multiple environmental systems, among which the school environment represents a central microsystem directly influencing behavior (Bronfenbrenner, 2000). During adolescence, time spent in family contexts gradually decreases, while school becomes the primary environment for social interaction and development (Yüksel Dogan and Demircioglu, 2025).

School climate refers to the overall quality and atmosphere of school life, including interpersonal relationships, teaching practices, institutional norms, and organizational structures (Petrie, 2014). A positive school climate, characterized by supportive teacher–student relationships, peer cohesion, and fair disciplinary practices can foster emotional security, strengthen social bonds, and promote positive behavioral norms, thereby reducing aggressive behavior (Akman, 2021; Petrie, 2014; Kuzhiyengal Mambra and Kotian, 2024).

Conversely, a negative school climate, marked by poor relationships, lack of support, or perceived unfairness, may increase adolescents' frustration, insecurity, and hostility, ultimately contributing to aggressive behavior (Barth et al., 2004; Akman, 2021). These findings highlight the importance of school climate as a key environmental factor influencing adolescent behavior.

### 1.2 Mediating role of friendship quality

While ecological systems theory emphasizes environmental influences (Bronfenbrenner, 2000), from an interpersonal and developmental perspective, peer relationships, particularly friendships, play a central role in adolescents' social and emotional development (Cheng and Jiao, 2024). During this stage, friendships become increasingly important sources of emotional support, identity formation, and social learning.

Friendship quality is a multidimensional construct that includes positive aspects (e.g., intimacy, support, companionship, and trust) as well as negative aspects (e.g., conflict and betrayal; Preddy and Fite, 2012). High-quality friendships provide adolescents with emotional security, social support, and opportunities to develop interpersonal skills, which can promote adaptive behavior and reduce aggression (Criss et al., 2002; Preddy and Fite, 2012). Conversely, low-quality or conflictual friendships may undermine trust and increase the likelihood of maladaptive behaviors, including aggression (Malti et al., 2015). In the Inner Mongolia context, where some adolescents in rural and pastoral areas experience parental absence due to labor migration, peer relationships within schools may increasingly substitute for family-based support, further highlighting the importance of friendship quality (Wu and Wang, 2021). Furthermore, Mongolian herdsmen families are typically dispersed across remote areas with relatively limited contact with the outside world, making school-based friendships a central source of social interaction beyond the family (Wu and Wang, 2021).

Importantly, the school environment may influence adolescents' aggressive behavior indirectly through its impact on friendship quality. A supportive school climate can facilitate positive peer interactions and the development of high-quality friendships, which in turn provide protective social resources that reduce aggressive tendencies (Cheng and Jiao, 2024). In contrast, negative school environments may hinder the formation of supportive peer relationships (Cheng and Jiao, 2024), thereby increasing vulnerability to aggression.

From an interpersonal process perspective, friendship quality represents a key mechanism linking environmental influences to behavioral outcomes. However, it should be noted that friendship quality is likely one of several mechanisms through which school climate affects adolescent behavior, rather than the sole explanatory factor.

### 1.3 Moderating role of psychological resilience

The direct and indirect pathways through which perceived school climate influences adolescent aggressive behavior may be moderated by other individual factors. In this study, psychological resilience is conceptualized as a dynamic adaptive process encompassing multiple protective factors, including positive cognition, positive emotions, and problem-solving abilities, which enable adolescents to respond effectively to environmental challenges (Fletcher and Sarkar, 2013). Importantly, contemporary resilience frameworks conceptualize resilience not as a fixed personality trait but as a context-dependent and dynamic process that develops through interactions between individuals and their environments, thereby aligning closely with ecological perspectives on development (Kumpfer, 1999).

According to psychological resilience models, individuals who are able to mobilize higher levels of adaptive resilience processes can more effectively utilize internal and external resources to buffer the impact of risk factors, such as negative school environments, thereby reducing the likelihood of negative behaviors (Kumpfer, 1999). In other words, psychological resilience may buffer the adverse impact of a negative school environment on adolescents' aggressive behavior. Even in unfavorable school contexts, adolescents who can effectively mobilize resilience processes, such as emotion regulation, are less likely to resort to aggression as a coping strategy (Saraswati and Suleeman, 2018). Empirical evidence suggests that psychological resilience enhances adolescents' positive perceptions of the school environment and mitigates aggressive tendencies (Dias and Cadime, 2017). Furthermore, Grol and De Raedt (2018) found that psychological resilience moderates the relationship between environmental perceptions and aggressive behavior. From an interpersonal regulatory perspective, resilience may influence how adolescents interpret and respond to social stressors within the school environment, thereby shaping whether environmental challenges translate into maladaptive behavioral outcomes such as aggression (Grol and De Raedt, 2018; Sullivan, 2013). This process may be particularly salient in the Inner Mongolia context, where adolescents may need to navigate both boarding school environments and intercultural peer interactions, potentially placing greater demands on adaptive resilience processes.

In addition to moderating the direct association between perceived school climate and aggressive behavior, psychological resilience may also influence the mediating process of friendship quality. From the perspective of the psychological resilience model, resilience functions as a protective process that buffers individuals against adverse environmental influences (Kumpfer, 1999). Research indicates that individuals with high psychological resilience are better able to counteract the detrimental effects of a negative school climate on friendship quality, or conversely, to strengthen the beneficial effects of a positive school climate (Fang et al., 2025). This perspective is also supported by interpersonal theories of development (Sullivan, 2013), which suggest that individual differences in emotional regulation and social competence shape the formation and maintenance of peer relationships (Wang et al., 2017). Adolescents with higher resilience may be better equipped to initiate, maintain, and repair friendships, even in less supportive environments, thereby sustaining higher-quality peer interactions.

According to the psychological resilience model, protective factors enable individuals to mobilize internal and external resources to achieve positive social adaptation, thereby reducing the likelihood of negative behaviors (Kumpfer, 1999). During adolescence, when peers become increasingly central to social life, resilient individuals tend to engage in more adaptive social interactions and experience fewer behavioral problems (Zhang et al., 2023). High-quality friendships provide adolescents with valuable resources, such as social support, prosocial role models, and strategies for problem-solving (Bukowski et al., 1994; Wang et al., 2021). Adolescents who can effectively mobilize resilience processes are better able to utilize these friendships (Fu et al., 2023; Graber et al., 2016). For instance, when facing conflicts or challenges, such adolescents are more likely to seek support from peers and apply strategies learned through these relationships to regulate their emotions and resolve difficulties, thereby reducing the likelihood that stress will manifest as aggressive behavior (Bokhorst et al., 2010). Through these supportive relationships, adolescents can better regulate their emotions and behaviors, reducing the likelihood of aggression (Yan et al., 2025; Waldrip et al., 2008). Moreover, the coexistence of high friendship quality and strong psychological resilience may exert an interactive protective effect on adolescents' maladaptive behaviors (Li and Si, 2024). Empirical findings further demonstrate that resilience promotes adolescents' capacity to maintain high-quality friendships in stressful contexts, thereby decreasing the occurrence of conflictual or aggressive behaviors (Fu et al., 2023). Taken together, these perspectives suggest that psychological resilience may not only buffer environmental risks but also shape how effectively adolescents translate interpersonal resources into adaptive behavioral outcomes.

In summary, this study constructs a moderated mediation model to examine the influence of perceived school climate on adolescent aggressive behavior and to elucidate the underlying mechanisms linking these variables. By integrating resilience frameworks with interpersonal and ecological perspectives, this approach provides a more comprehensive conceptual basis for understanding how individual adaptive capacities condition both environmental and relational influences on adolescent behavior.

### 1.4 Present study and hypotheses

Building on ecological systems theory, interpersonal perspectives, and resilience frameworks, this study constructs a moderated mediation model to examine the influence of perceived school climate on adolescent aggressive behavior.

Despite the growing body of research on school climate and adolescent behavior, the present study offers several important contributions. First, rather than examining direct associations alone, this study integrates environmental, interpersonal, and individual factors within a single moderated mediation framework, providing a more comprehensive understanding of the mechanisms underlying adolescent aggressive behavior. Second, by conceptualizing friendship quality as one of multiple interpersonal pathways, rather than a dominant explanatory factor, this study offers a more nuanced interpretation of how peer relationships function within broader ecological systems. Third, the inclusion of psychological resilience as a moderating factor extends existing research by highlighting how individual adaptive capacities may condition both direct and indirect environmental effects. Finally, by drawing on data from the Inner Mongolia Autonomous Region and including students across primary, middle, and high school levels, this study contributes empirical evidence from an underrepresented cultural and developmental context, thereby enriching the global literature on adolescent development and school-based behavioral outcomes.

Based on the theoretical and empirical literature, the following hypotheses are proposed:

**H1:** Perceived school climate significantly negatively predicts adolescent aggressive behavior.

**H2:** Friendship quality mediates the relationship between perceived school climate and adolescent aggressive behavior.

**H3a:** Psychological resilience moderates the relationship between perceived school climate and adolescent aggressive behavior.

**H3b:** Psychological resilience moderates the relationship between perceived school climate and friendship quality.

**Figure 1:**
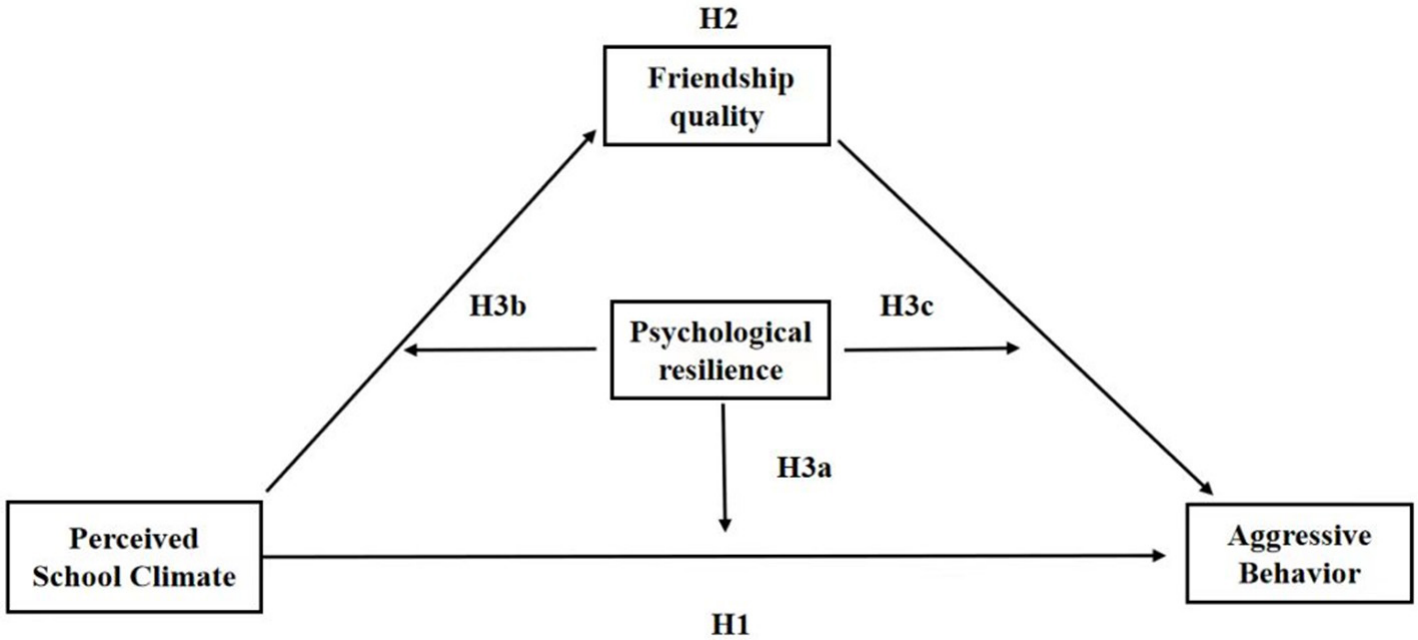
Moderated mediation model.

**H3c:** Psychological resilience moderates the relationship between friendship quality and adolescent aggressive behavior. Figure 1 shows the moderated mediation model.

## 2 Methods

### 2.1 Participants and procedure

This study uses baseline data from a larger longitudinal project at the Research Center for Student Bullying Prevention and Control in the Inner Mongolia Autonomous Region, China. The project involves a series of investigations, with a follow-up survey planned with the same cohort of students 1 year later. The Inner Mongolia Autonomous Region is a multi-ethnic area characterized by collectivist cultural influences, a diverse ethnic composition (e.g., Mongolian and Han populations), and a geographically dispersed population (Shen and Wang, 2025; Wu and Wang, 2021; Zhang, 2026). In many areas, boarding schools are common, and parental migration for work may lead adolescents to rely more heavily on peer relationships within school settings. These contextual characteristics provide a relevant setting for examining school climate and adolescent behavior.

This study employed a cluster sampling method to conduct a large-scale survey in the Inner Mongolia Autonomous Region, China, from June 9 to June 13, 2025. A total of 36 public schools were invited to participate. The selection of schools was informed by a large-scale survey on bullying conducted by a research team from Capital Normal University. Based on this survey, 36 general and vocational schools were purposively selected from 12 regions identified as having relatively high reported incidences of bullying. First, purposive sampling was used to select 12 high schools, 12 middle schools, and 12 primary schools as the primary sampling units across the region, ensuring representation across different educational stages. Subsequently, within each sampled school, three classes were randomly selected from the target grade levels (high school: grades 10 and 11; middle school: grades 1 and 2; primary school: grades 4 and 5) as the secondary sampling units. These specific grade levels were selected to align with the broader longitudinal project and to enhance the comparability of subsequent follow-up data.

Each school designated a liaison (typically a teacher or administrative staff member with both instructional and coordination responsibilities) to facilitate communication and assist in organizing the data collection process. No additional school-level information (e.g., school size, resources, or institutional characteristics) was collected, as the study focused primarily on student-level perceptions and behaviors, and limiting such data helped reduce administrative burden and improve participation rates.

Self-report questionnaires were administered on-site under the supervision of trained school personnel, including teachers and principals, who assisted in distributing and collecting paper-based instruments. In total, 5,870 students aged 8 to 20 years participated in the survey. After excluding questionnaires that were incomplete or showed evidence of careless or invalid responses, 4,991 valid responses were retained, yielding an effective response rate of 85.02%. Among the valid respondents, 2,308 (46.24%) were males and 2,683 (53.75%) were females. Participants' ages ranged from 8 to 20 years (M = 14.36, SD = 2.65).

Ethical approval for this research was obtained from the Ethics Committee of Medicine and Life Sciences of Inner Mongolia University for Nationalities (Approval No.: NMD-RT-2025-06-04). The ethical approval obtained corresponds to the broader research project, from which this study draws on a subset of variables relevant to the current research objectives.

Permission to conduct the study was granted by the local education authorities as well as the administrators and teachers at the participating schools. Parents or legal guardians of all participants were informed about the purpose and procedures of the study, and participation was entirely voluntary. Written informed consent was obtained from all participants prior to data collection. All research procedures adhered to the ethical principles outlined in the National Research Council guidelines and the 1964 Declaration of Helsinki and its subsequent amendments or comparable ethical standards.

### 2.2 Instruments

#### 2.2.1 Perceived school climate scale for adolescents

The Adolescent Perceived School Climate Scale used in this study was adapted from the scale originally developed by Jia et al. (2009) and later revised by Zheng et al. (2015). To ensure the accuracy and reliability of the instrument, a translation–back-translation procedure was carried out independently by two bilingual researchers. Based on this process, the questionnaire was further revised to reflect the local educational context of Chinese adolescents. In addition, three experts in educational psychology and adolescent development were invited to assess the content validity of the scale, and a pilot test was conducted at Yifu Primary School to evaluate the scale's operability and applicability and identify any potential issues. Ultimately, the scale was found suitable for large-scale implementation. The Adolescent Perceived School Climate Scale consists of three dimensions: opportunity for autonomy, student support, and teacher support, comprising 25 items (*e.g., I can share my problems and challenges with my teacher; Students use derogatory nicknames for one another*.), including seven reverse-scored items. Each item is rated on a 4-point Likert scale ranging from 1 (“never”) to 4 (“always”), with higher total scores indicating a more positive perception of school climate. In the present study, the scale demonstrated good internal consistency, with a Cronbach's alpha coefficient of 0.825.

#### 2.2.2 Friendship quality scale

The Friendship Quality Scale developed by Parker and Asher (1993) was employed in this study to assess the quality of children's friendships. The Chinese version of the scale, revised by Zou Han and Wan Jingjing, has demonstrated strong reliability and validity in previous research. To further examine its applicability and operability and identify any potential issues, a pilot test was conducted at Yifu Primary School. Ultimately, the scale was found suitable for large-scale implementation. The original scale comprises 40 items (*e.g., We often discuss the problems we're facing together; This friend makes me feel that some of my ideas are quite good*.), from which 18 items with the highest factor loadings were selected for use in this study. The results showed χ^2*df*^ = 4.884, RMSEM = 0.028, CFI = 0.988, TLI = 0.976, and SRMR = 0.012. A follow-up validation factor analysis revealed that, with the exception of factor 5, the combined confidence (CR) of the factors was between 0.871 and 0.989 and the mean variation extraction (AVE) was between 0.659 and 0.967. This indicated that the scale with the chosen 18 items had good reliability and convergent validity. These items cover six dimensions of friendship quality: affirmation and care, conflict and betrayal, conflict resolution strategies, help and guidance, companionship and recreation, and intimate disclosure and communication. Responses were rated on a 5-point Likert scale ranging from 1 (“completely disagree”) to 5 (“completely agree”), with higher scores indicating higher levels of friendship quality. In this study, the scale demonstrated good internal consistency, with a Cronbach's alpha coefficient of 0.888. The confirmatory factor analysis was conducted following established structural equation modeling procedures (Byrne, 2010).

#### 2.2.3 Psychological resilience scale

The Adolescent Psychological Resilience Scale used in this study was adapted from Hu and Gan (2008). To ensure the accuracy and reliability of the instrument, two bilingual researchers independently conducted a translation–back-translation procedure on the original scale. Based on this process, the questionnaire was localized and revised to align with the educational and cultural context of Chinese adolescents. Three experts in psychology and education were invited to evaluate the content validity of the adapted version, and a pilot test was administered at Yifu Primary School to assess its operability and applicability and identify any potential issues. Ultimately, the scale was found suitable for large-scale implementation. The scale comprises 27 items (*e.g., Failures always leave me feeling disheartened; When I'm facing difficulties, I make an effort to talk to someone about it*.), including 11 reverse-scored items. Each item is rated on a 5-point Likert scale ranging from 1 (“completely inconsistent”) to 5 (“completely consistent”), with higher scores indicating higher levels of psychological resilience. In the present study, the scale demonstrated acceptable internal consistency, with a Cronbach's alpha coefficient of 0.782.

#### 2.2.4 Aggressive behavior scale

The Chinese version of the Aggression Questionnaire, developed by Buss and Perry (1992) and revised by Liu et al. (2009) was employed in this study. The scale is appropriate for use among elementary, junior high, and high school students. To examine its applicability and operability, a pilot test was conducted at Yifu Primary School. The questionnaire comprises 4 dimensions: physical aggression, verbal aggression, anger, and hostility, with 29 items (*e.g., Some of my friends think that I have a short temper; I will employ force if necessary to protect my rights*.), including three reverse-scored items. Each item is rated on a 5-point Likert scale ranging from 1 (“completely inconsistent”) to 5 (“consistent”), with higher total scores indicating stronger tendencies toward aggressive behavior. In this study, the scale demonstrated good internal consistency, with a Cronbach's alpha coefficient of 0.848.

### 2.3 Research procedure

Data analysis was conducted using SPSS 24.0. Descriptive statistics and correlation analyses were first performed, followed by mediation and moderation effect tests using the PROCESS 3.3 macro developed by Hayes (Model 4 for mediation and Model 59 for moderated mediation). To assess potential common method bias, Harman's single-factor test was conducted. Multicollinearity among predictor variables was examined using variance inflation factor (VIF) values.

Considering that non-normal data distribution or heterogeneity of variance during parameter estimation may increase the likelihood of Type I and Type II errors, this study employed the bias-corrected nonparametric percentile bootstrap method to test the significance of regression coefficients, based on Erceg-Hurn and Mirosevich (2008). Specifically, 5,000 bootstrap samples were drawn to estimate the standard errors of the parameter coefficients and to generate 95% bootstrap confidence intervals. A confidence interval that did not include zero, indicating a statistically significant effect (Wen and Ye, 2014).

### 2.4 Common method bias and multicollinearity tests

All data in this study were collected through self-report questionnaires; therefore, potential common method bias was examined (Podsakoff et al., 2003). Harman's single-factor test was conducted to assess this issue. The results of the unrotated exploratory factor analysis identified 16 factors with eigenvalues greater than 1, and the first factor accounted for 17.578% of the total variance, which is well below the critical threshold of 40% (Podsakoff et al., 2003). These results suggest that common method bias was not a serious concern. Furthermore, multicollinearity among the predictor variables was examined using the VIF in SPSS. The tolerance values for all predictor variables were 0.959, 0.977, and 0.944, all exceeding the recommended minimum of 0.10, while the corresponding VIF values were 1.043, 1.024, and 1.060, all below the cutoff of 5. These results indicate that multicollinearity was not present among the predictor variables. Thereby, the findings demonstrate that the data used in this study possess strong reliability and stability (Hair et al., 2019).

## 3 Results

### 3.1 Descriptive statistics and correlation analysis

Descriptive statistics and correlation analyses for perceived school climate, friendship quality, aggressive behavior, and psychological resilience are presented in Table 1.

**Table 1:** Descriptive statistics and correlation analysis among variables.

Variables	M ± SD	Minimum value	Maximum value	1	2	3	4
1. Perceived school climate	23.44 ± 3.335	7	28	1			
2. Friendship quality	57.59 ± 14.805	78	90	0.074^**^	1		
3. Aggressive behavior	29.78 ± 11.764	32	131	−0.267^**^	−0.069^**^	1	
4. Psychological resilience	57.90 ± 13.181	0	51	0.198^**^	0.145^**^	−0.433^**^	1

^**^p < 0.01.

Perceived school climate was significantly positively correlated with friendship quality (*r* = 0.074, *p* < 0.01) and psychological resilience (*r* = 0.198, *p* < 0.01), and significantly negatively correlated with aggressive behavior (*r* = −0.267, *p* < 0.01). Friendship quality was significantly negatively correlated with aggressive behavior (*r* = −0.069, *p* < 0.01) and significantly positively correlated with psychological resilience (*r* = 0.145, *p* < 0.01). Aggressive behavior was significantly negatively correlated with psychological resilience (*r* = −0.433, *p* < 0.01).

Although these correlations were statistically significant, some associations, particularly those involving friendship quality (e.g., *r* = 0.074 and *r* = −0.069), were relatively weak in magnitude and should be interpreted with caution.

### 3.2 Mediation effect test

The mediating effect of friendship quality was examined using the PROCESS macro (Model 4). As shown in Table 2, perceived school climate significantly predicted aggressive behavior (β = −0.277, *p* < 0.001). The indirect effect of perceived school climate on aggressive behavior through friendship quality was statistically significant (indirect effect = −0.003, SE = 0.001, 95% CI [−0.006, −0.001]). The proportion of the indirect effect accounted for 1.44% of the total effect, indicating a relatively small mediating effect. These findings suggest that friendship quality partially mediates the relationship between perceived school climate and aggressive behavior, although the magnitude of this mediation is modest.

**Table 2:** Friendship quality mediating effect test.

Path	Effect size	SE	95% CI	Relative mediating effect
Perceived school climate → aggressive behavior	−0.277	0.013	−0.304~-0.251	98.56%
Perceived school climate → friendship quality → aggressive behavior	−0.003	0.001	−0.006~-0.001	1.44%
Total effect	−0.281	0.014	−0.301~-0.254	100%

### 3.3 Moderation effect test

Using IBM SPSS Statistics 24 and the PROCESS macro 3.3 (Model 59), this study examined the moderating effect of psychological resilience across the three pathways of the mediation model. Table 3 presents the result. The interaction between perceived school climate and psychological resilience did not significantly predict friendship quality (β = −0.003, *p* > 0.05). However, the interaction between perceived school climate and psychological resilience significantly predicted aggressive behavior (β = 0.038, *p* < 0.01). Furthermore, the interaction between friendship quality and psychological resilience significantly predicted aggressive behavior (β = −0.029, *p* < 0.05). These findings indicate that psychological resilience significantly moderates the relationships between perceived school climate and aggressive behavior, and between friendship quality and aggressive behavior, but not the relationship between perceived school climate and friendship quality.

**Table 3:** Moderating role of psychological resilience in the mediating model of perceived school climate on aggressive behavior.

Variables	Equation 1: friendship quality			Equation 2: aggressive behavior		
	β	***t***	**95% CI**	β	***t***	**95% CI**
Perceived school climate	0.039	2.717^**^	[0.011, 0.068]	−0.194	−14.949^***^	[−0.219, −0.168]
Psychological resilience	0.139	9.579^***^	[0.110, 0.167]	0.386	−29.418^***^	[−0.411, −0.360]
Perceived school climate × Psychological resilience	−0.003	−0.239	[−0.030, 0.024]	0.038	3.060^**^	[0.014, 0.062]
Friendship quality				0.011	0.795	[−0.015, 0.036]
Friendship quality × psychological resilience				−0.029	−2.468^*^	[−0.053, −0.006]
Sex	0.093	6.604^***^	[0.065, 0.120]	0.035	2.752^**^	[0.010, 0.059]
Age	−0.023	−1.619	[−0.051, 0.005]	−0.055	−4.333^***^	[0.030, 0.080]
*R* ^2^	0.032		0.228			
*F*	33.236		210.342			

^*^p < 0.05, ^**^p < 0.01, ^***^p < 0.001.

### 3.4 Simple slope analysis

**Figure 2:**
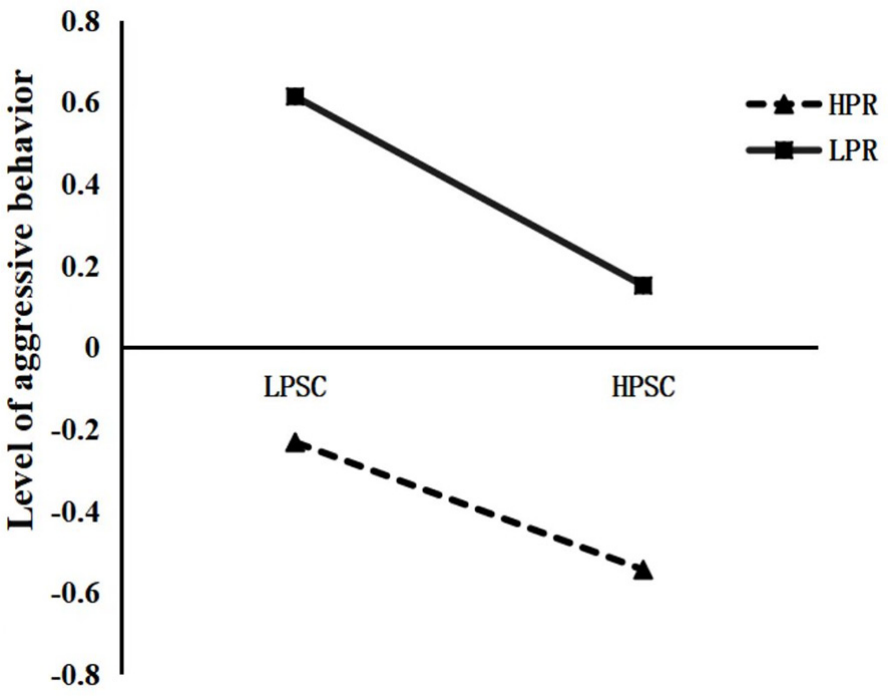
Interactive effect of perceived school climate and psychological resilience on aggressive behavior. LPSC, low perceived school climate; HPSC, high perceived school climate; HPR, high psychological resilience; LPR, low psychological resilience.

**Figure 3:**
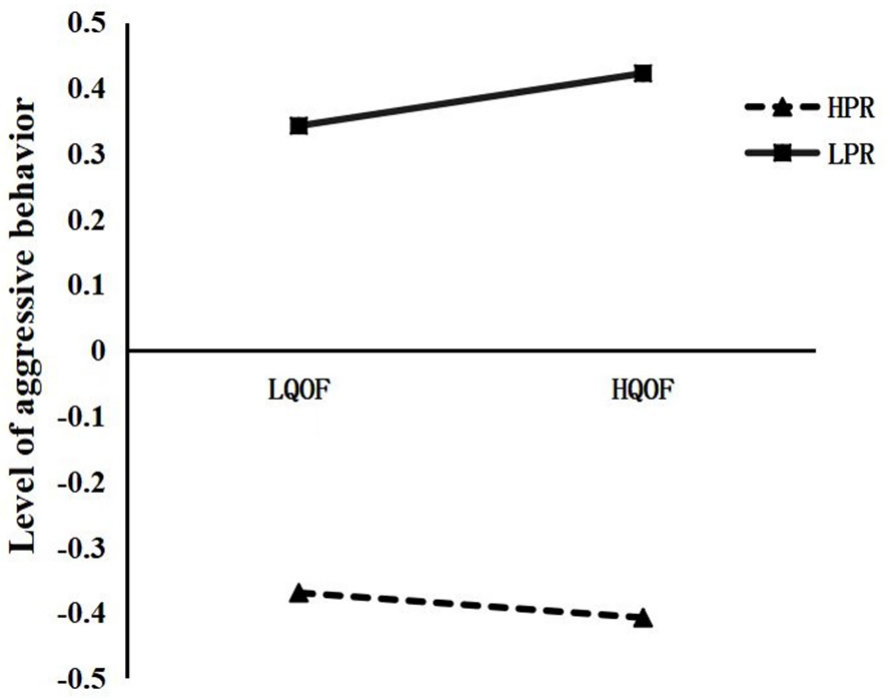
Interactive effect of friendship quality and psychological resilience on aggressive behavior. LQOF, low quality of friendships; HQOF, high quality of friendships; HPR, high psychological resilience; LPR, low psychological resilience.

To further examine the moderating effects, participants were divided into high and low psychological resilience groups (±1 SD), and simple slope analyses were conducted. The results are presented in Figures 2, 3. As shown in Figure 2, perceived school climate was significantly negatively associated with aggressive behavior in both low-resilience (bsimple = −0.231, *p* < 0.001) and high-resilience groups (bsimple = −0.156, *p* < 0.001), with a stronger effect observed in the low-resilience group.

As shown in Figure 3, friendship quality significantly negatively predicted aggressive behavior in the low-resilience (bsimple = −0.040, *t* = 1,976, *p* < 0.05). This relationship was not statistically significant in the high-resilience (bsimple = −0.019, *t* = −1.261, *p* = 0.208); however, their overall aggressive behavior levels were substantially lower than the risk threshold.

## 4 Discussion

### 4.1 Overview of findings

This study examined the relationships among perceived school climate, friendship quality, psychological resilience, and adolescent aggressive behavior using a large sample of 4,991 students from 36 primary, middle, and high schools in the Inner Mongolia Autonomous Region of China. By integrating environmental, interpersonal, and individual factors, the findings provide insight into how multiple levels of influence are associated with adolescent aggression.

Importantly, these findings extend existing research by examining these associations within a large and developmentally diverse sample spanning primary, middle, and high school students, thereby offering a broader perspective on how school climate relates to aggression across different developmental stages. In addition, by situating these relationships within a contemporary context, the study responds to increasing concerns about adolescent behavior in rapidly changing social environments, including the influence of digital communication and evolving peer interaction patterns.

The results indicate that perceived school climate is significantly associated with adolescent aggressive behavior, with more positive school environments linked to lower levels of aggression. This finding is consistent with previous research showing that a supportive school climate enhances adolescents' sense of security and belonging, thereby reducing the likelihood of aggressive behavior (Cohen, 2009; Thomas, 2019). One possible mechanism underlying this relationship is that supportive school environments reduce interpersonal stress and foster emotional regulation, which in turn lowers aggressive responses (Way et al., 2007; Eddles-Hirsch et al., 2012). Conversely, a poor school environment—characterized by low classroom quality, insufficient teacher support, weak peer relationships, or negative teacher–student interactions—may undermine adolescents' emotional stability and increase the risk of aggressive behavior (Wang et al., 2017).

From the perspective of ecological systems theory, adolescent behavior arises from dynamic interactions between individuals and their environments (Bronfenbrenner, 2000). A supportive school environment provides resources, structure, and emotional security that facilitate adaptive development and reduce the likelihood of maladaptive behaviors such as aggression.

### 4.2 Mediating role of friendship quality

In this study, although the indirect effect of school climate via friendship quality accounted for a relatively small proportion of the total effect, this finding is consistent with ecological models of development, which suggest that no single interpersonal mechanism fully explains complex behaviors such as aggression. Instead, friendship quality represents one of multiple concurrent social processes through which school climate may be associated with adolescent behavior (Gendron et al., 2011), supporting H2. The relatively small mediating effect (1.44%) indicates that this pathway is modest in magnitude and should be interpreted with caution, as adolescent aggressive behavior is likely influenced by multiple contextual and individual factors (e.g., family environment, personality traits, and media exposure) (Estévez López et al., 2008; Kuzhiyengal Mambra and Kotian, 2024; Nesi et al., 2018; Zhang et al., 2022; He and Wen, 2025).

This modest effect size is consistent with contemporary research suggesting that adolescent friendships are increasingly shaped by complex and hybrid (online–offline) interaction contexts, where peer influence operates alongside multiple competing social inputs (Nesi et al., 2018; Prinstein and Giletta, 2020). As such, friendship quality may function as one contributing pathway rather than a dominant mechanism, particularly in modern social environments characterized by digital communication and broader peer networks (Prinstein and Giletta, 2020).

This suggests that a positive school environment can facilitate the development of high-quality friendships, which, in turn, may reduce aggressive behavior by enhancing adolescents' sense of security and competence (Zhang et al., 2022). One possible mechanism is that supportive peer relationships provide emotional validation and reduce interpersonal stress, thereby lowering the likelihood of aggressive responses. This result is consistent with ecological systems theory, which posits that environmental factors, such as school climate, shape individual development through dynamic interactions with the social context (Bronfenbrenner, 2000).

Specifically, schools with limited resources or high levels of stress may heighten competition and interpersonal tension among adolescents, increase feelings of insecurity, and undermine the quality of peer relationships, potentially contributing to aggressive behavior (Reynolds, 2002). Conversely, a supportive school environment fosters caring and mutually beneficial peer relationships, providing adolescents with both emotional and instrumental support. This environment enhances the quality of friendships, which, in turn, can reduce aggressive behavior (Cheng and Jiao, 2024).

Adolescents with high-quality friendships are more likely to receive positive reinforcement and inclusive support from peers, which strengthens emotional security, mitigates emotional distress, promotes socially adaptive behavior, and may decrease the likelihood of aggressive tendencies (Yang et al., 2020; Preddy and Fite, 2012).

### 4.3 Moderating role of psychological resilience

This study found that psychological resilience significantly and negatively moderated the predictive effect of perceived school climate on adolescent aggressive behavior, supporting H3a. Psychological resilience, conceptualized as a dynamic process encompassing protective factors, may enhance adolescents' adaptability through emotion regulation, reduce the likelihood of using negative behaviors to address challenges, and enable them to cope more effectively with high-risk school environments (Dias and Cadime, 2017; Richardson, 2002).

Compared with individuals with low psychological resilience, the predictive effect of perceived school climate on aggressive behavior appears to differ across levels of resilience (Zhang and Li, 2025). According to the psychological resilience model, when adolescents encounter adverse school environments, those who can effectively mobilize adaptive resilience processes may be more likely to adjust their perceptions, apply positive coping strategies, and reduce aggressive behavior over time (Kumpfer, 1999). Meanwhile, adolescents with high psychological resilience may have a greater capacity to buffer the negative impact of adverse school environments, thereby mitigating maladaptive behaviors (Luthar et al., 2000).

Second, this study found that the predictive effect of perceived school climate on friendship quality was not moderated by psychological resilience, and thus H3b was not supported. From the perspective of the psychological resilience model, resilience functions as a protective process, with individuals demonstrating stronger emotion regulation and problem-solving abilities (Kumpfer, 1999).

When faced with conflicts or challenges, these adolescents may leverage supportive peer relationships to gain emotional security and obtain constructive guidance, rather than resorting to aggressive responses (Wang et al., 2021). Conversely, adolescents with lower levels of resilience may struggle to utilize high-quality peer relationships effectively, limiting their capacity to regulate impulses and control aggressive behavior (Liu and Feng, 2024; Wang et al., 2021). These findings help explain why psychological resilience does not appear to moderate the predictive effect of the school environment on friendship quality.

Finally, the study found that psychological resilience moderated the latter stage of the indirect pathway in which perceived school climate influences aggressive behavior through friendship quality, supporting H3c. Specifically, among adolescents with low psychological resilience, higher friendship quality significantly and negatively predicted aggressive behavior.

However, adolescents with low psychological resilience may be less able to utilize high-quality friendships and peer resources to seek support, thereby diminishing the protective effect of these relationships (Patenteu et al., 2024). In contrast, among adolescents with high psychological resilience, the predictive effect of friendship quality on aggressive behavior was not statistically significant, yet their overall aggression levels were substantially lower.

It should also be noted that some of these interaction effects, although statistically significant, are relatively modest in magnitude and should be interpreted with appropriate caution.

Taken together, these findings contribute to the literature by demonstrating that individual adaptive capacities, such as psychological resilience, may condition both direct and indirect associations between environmental and interpersonal factors and adolescent behavior. This integrative perspective moves beyond traditional single-path models and highlights the importance of examining how multiple systems interact in shaping behavioral outcomes.

The findings of this study have both theoretical and practical implications. From a theoretical perspective, this study extends ecological systems theory by illustrating how the microsystem of the school environment, peer relationships (friendship quality), and the individual-level factor of psychological resilience are jointly associated with adolescent aggressive behavior (Bronfenbrenner, 2000). It provides empirical evidence on the interplay among environmental, interpersonal, and individual factors. In addition, the findings align with psychological resilience frameworks, suggesting that resilience may not only mitigate the impact of adverse conditions but also influence how individuals utilize available social resources (Kumpfer, 1999).

These findings may be particularly relevant in the Chinese cultural context, where interpersonal harmony and group cohesion are emphasized (Yang, 2012; Chen and Zhang, 2010). In such contexts, school climate and peer relationships may play a particularly important role in shaping adolescents' behavioral and emotional development (Banerjee et al., 2025).

From a practical perspective, mitigating adolescent aggression may require a comprehensive approach that addresses multiple levels of influence. Schools should cultivate supportive and inclusive environments that foster positive teacher–student and peer relationships (Akman, 2021). For example, interventions such as cooperative learning strategies, peer mentoring programs, and social-emotional learning initiatives may help improve friendship quality and promote prosocial behavior. At the same time, strengthening adolescents' psychological resilience through interventions such as emotional regulation training, stress management programs, and coping skills development may further reduce aggressive behavior and promote adaptive development (Troy et al., 2023).

These findings are particularly relevant in contemporary educational contexts, where schools are increasingly required to address both traditional forms of aggression and emerging challenges related to peer interaction in digital and hybrid social environments. By identifying multiple points of intervention, school climate, peer relationships, and individual resilience, this study offers a more comprehensive basis for designing targeted and context-sensitive prevention strategies.

Overall, by integrating environmental, interpersonal, and psychological factors within a unified framework, this study offers a more comprehensive perspective on the factors associated with adolescent aggression and provides useful directions for both future research and practical intervention.

### 4.4 Limitations and future research

Despite the contributions of this study, several limitations should be acknowledged.

First, the sample of 4,991 adolescents was drawn exclusively from 36 public schools in the Inner Mongolia Autonomous Region of China. Although this provides valuable insight into an understudied regional context, the findings may not be fully generalizable to other regions or cultural settings. Differences in educational systems, socioeconomic conditions, and cultural norms may influence the relationships observed in this study. Future research should include more diverse and representative samples across multiple regions and, where possible, conduct cross-cultural comparisons to enhance external validity.

Second, this study employed a cross-sectional design, which limits the ability to draw causal inferences among perceived school climate, friendship quality, psychological resilience, and aggressive behavior. While the proposed model is theoretically grounded, the directionality of these relationships cannot be definitively established. In particular, although this study assumes that school climate influences adolescent aggressive behavior, the reverse relationship is also plausible—adolescents' aggressive behavior may negatively contribute to or shape the overall school climate. For example, it is also plausible that adolescents' aggressive behavior may influence their perception of school climate or the quality of their peer relationships. Future studies are encouraged to adopt longitudinal or experimental designs to better examine causal pathways and developmental processes over time.

Third, although this study identified friendship quality as a mediating variable, the indirect effect size was relatively small (1.44% of the total effect). This suggests that friendship quality represents only one of multiple mechanisms through which school climate may be associated with aggressive behavior. Other potential mediating processes, such as emotional regulation, self-control, social competence, or perceived teacher support, were not examined in the present study and may play a more substantial role. Future research should explore more comprehensive models that incorporate multiple mediators and their potential interactions.

Fourth, several potential confounding variables were not included in the analysis. Factors such as family environment (e.g., parenting style, family conflict), socioeconomic status, academic pressure, and exposure to media or online environments may also influence adolescent aggressive behavior. The omission of these variables may limit the explanatory power of the model. Future studies should incorporate these contextual and individual-level factors to provide a more comprehensive understanding of adolescent behavior.

Fifth, all data were collected through self-report questionnaires, which may introduce common method bias and subjective reporting errors. Although statistical tests suggested that common method bias was not a serious concern, self-reported data may still be influenced by social desirability or recall bias. Future research should consider using multi-informant approaches, including teacher reports, peer evaluations, or behavioral observations, as well as objective or performance-based measures where possible.

Finally, although this study is part of a larger longitudinal research project, only the first wave of data was analyzed. Future analyses using follow-up data from the same cohort will allow for a more robust examination of developmental trajectories and causal relationships among the variables. Such longitudinal evidence would provide stronger support for the proposed theoretical framework.

In summary, while this study provides initial evidence on the relationships among perceived school climate, friendship quality, psychological resilience, and adolescent aggressive behavior, further research is needed to address these limitations and to develop a more comprehensive and causally robust understanding of these processes.

## 5 Conclusion

This study suggests that adolescents who perceive a more positive school climate tend to report lower levels of aggressive behavior, both directly and indirectly through higher friendship quality. Furthermore, psychological resilience was found to moderate these relationships, potentially buffering the negative effects of less supportive school environments and lower-quality peer interactions. These findings integrate insights from ecological systems theory and psychological resilience frameworks, indicating that adolescent aggression may be associated with the combined influence of environmental conditions, interpersonal dynamics, and individual characteristics. The results also suggest that friendship quality represents one of several pathways through which the school environment is related to adolescent behavior, while psychological resilience may function as a protective factor. It is important to note that some of the observed effects, particularly the mediating role of friendship quality, were relatively small, indicating that additional mechanisms may also contribute to these relationships. From a practical perspective, the findings highlight the importance of fostering supportive and inclusive school environments that promote positive teacher–student and peer relationships. Interventions aimed at strengthening adolescents' emotional resilience, such as social-emotional learning, peer mentoring, and stress management programs, may be beneficial in reducing aggressive behavior. In summary, by integrating environmental and psychological factors within a unified framework, this study provides useful insights into the factors associated with adolescent adjustment and offers directions for future research and practice.

## Data Availability

The data analyzed in this study is subject to the following licenses/restrictions: the data generated in this study can be obtained from the corresponding author upon request. Requests to access these datasets should be directed to Fei He. 739701092@qq.com.
